# Editorial: Peripheral Immunity in Parkinson's Disease: Emerging Role and Novel Target for Therapeutics

**DOI:** 10.3389/fneur.2019.01080

**Published:** 2019-10-15

**Authors:** Cristoforo Comi, Marco Cosentino, Rodrigo Pacheco

**Affiliations:** ^1^Neurology Unit, Department of Translational Medicine, Interdisciplinary Research Center of Autoimmune Diseases, Movement Disorders Center, University of Piemonte Orientale, Novara, Italy; ^2^Center of Research in Medical Pharmacology, University of Insubria, Varese, Italy; ^3^Laboratory of Neuroimmunology, Fundación Ciencia and Vida, Ñuñoa, Departamento de Ciencias Biológicas, Facultad de Ciencias de la Vida, Universidad Andres Bello, Santiago, Chile

**Keywords:** immunity, alpha-synucein, gut-brain axis, T cell, probiotics

Parkinson's disease (PD) is the second most common neurodegenerative disease, affecting up to 10 million people worldwide. PD has no cure yet, and patients rely only on symptomatic treatments. The hallmarks of PD are progressive loss of dopaminergic neurons in the substantia nigra, appearance of intracellular inclusions of aggregated α-synuclein (α-syn), called Lewy bodies (LB), and neuroinflammation resulting from microglia activation (1, 2). Understanding the causes of neurodegeneration in PD remains, so far, a challenging goal. Nevertheless, the recent identification of the involvement of peripheral immunity is raising increasing interest, as it may provide unprecedented opportunities to better understand PD pathogenesis, to identify clinically meaningful biomarkers and hopefully also novel therapeutic strategies (3, 4).

Besides a strong genetic association between the major histocompatibility class II locus and PD risk (5, 6), evidence supporting the role of peripheral immunity in PD include more rapid PD progression in the presence of a pro-inflammatory cytokine profile in the blood (7), a Th1-biased CD4+ T cell profile (8, 9), as well as an altered CD8+ T cell profile, with increased activation and reduced senescence markers (10). Remarkably, α-syn may trigger infiltration into the brain of T cells which in turn contribute to exacerbation of α-syn pathology, neurotoxicity and neurodegeneration (11–13), and the reported ability of T cells from PD patients to generate an autoimmune response to α-syn (14, 15) is even leading to reconsider PD as an autoimmune disorder (16).

Despite all this knowledge, however, the nature of immune dysregulation in PD, its relationship with neuroinflammation and neurodegeneration in the brain, the changes in peripheral immunity during disease progression, and whether targeting peripheral immunity may be beneficial to PD patients, all remain to be established. This Research Topic has been launched with the aim to collect high-quality and state-of-the-art articles covering all the aspects relevant to the relationship between immunity and neurodegeneration in PD, and bringing together research teams from different but synergistic scientific fields.

The 12 articles, seven review and five original articles, that were finally accepted for publication, offer the opportunity to explore the most relevant aspects of this emerging Research Topic. Of course, review articles discuss the contribution of immunity and inflammation to PD pathogenesis in a more general framework. This is the case of Caggiu et al., who focus on evidence linking infections and abnormal protein accumulation to immune system activation and critically discuss the possibility that autoimmunity may take part in PD pathogenesis. Fuzzati-Armentero et al. make an interesting parallel between PD patients and toxin-induced animal models, discussing differences and similarities in the context of neuroinflammation and immune responses, including the potential to guide novel therapeutic strategies. Troncoso Escudero et al. provide an updated perspective on the complex dynamics which orchestrate immune responses bothinside the central nervous system (CNS) and in the periphery. In this context, authors also discuss the potential targeting of astrocytes and microglia, as well as gut microbiome, with their therapeutic implications in PD.

García-González et al. review the involvement of stress signaling by the endoplasmic reticulum, which is crucially involved in protein aggregation and proteostasis dysfunction. Authors discuss how such signaling impacts on brain-associated immune cells and the possible implications to neuroinflammation and development of neurodegenerative diseases.

Scott et al. focus on the role of α-syn autoantibodies in PD, showing that there is weak evidence for an increase in α-syn auto-antibodies in PD patients particularly in early disease phase, but also underlining that more evidence is needed to support a robust relationship.

Finally, two reviews point to the role of T cells. Campos-Acuña et al. critically discuss the possibility that T cell driven inflammation, which has a crucial role in dopaminergic degeneration in PD, is triggered in the gut mucosa. Accordingly, they show how structural components of commensal bacteria and/or mediators produced by gut-microbiota, including short-chain fatty acids and dopamine, may affect the behavior of T cells, triggering the development of T cell responses against LB, initially confined to the gut mucosa but later extended to the brain. Storelli et al. summarize the current knowledge on the contribution of Th17 cells and IL-17 in PD, also assessing their therapeutic relevance. They underline that both animal and clinical studies are limited. Only a few studies provide mechanistic evidence and none of them investigates the eventual relationship between Th17/IL-17 and clinically relevant endpoints.

As regards original articles, Peralta Ramos et al. investigated the role of peripheral immune cells in the spreading of α-syn strains to the CNS. Authors provide evidence that α-syn administration in mice can induce microglia activation and leukocytes recruitment toward the CNS. Monocytes primed by intraperitoneal LPS administration internalize α-syn, with subsequent CNS dissemination. In addition, the α-syn ribbons strain determines differential recruitment of CD4+ and CD8+ T cells.

Elgueta et al. explore the role of dopamine receptor D3 (DRD3) signaling in peripheral blood CD4+ T cells from PD patients. They find that immune phenotypes favored by DRD3 signaling, namely Th1 and Th17 cells, are increased in the peripheral blood cells of PD patients compared to controls.

Further, they support their findings by selective DRD3-antagonism in this subset of lymphocytes in parkinsonian mice, obtaining a therapeutic effect on motor impairment.

Magistrelli et al. studied the effects of an *in vitro* challenge with probiotic bacterial strains to peripheral blood mononuclear cells (PBMCs) of PD patients and controls. All strains inhibited inflammatory cytokines and ROS production in both patients and controls, but most strikingly *Lactobacillus salivarius* and *acidophilus*. Furthermore, most strains restored the integrity of an artificial membrane model integrity and inhibited *Escherichia coli* and *Klebsiella pneumoniae* overgrowth. Finally, authors showed that the studied strains did not express tyrosine decarboxylase genes, which are known to decrease levodopa bioavailability.

Wijeyekoon et al. assessed monocyte functions in early-moderate PD compared to age and gender-matched controls. They found that PD monocytes display enhanced phagocytosis, but no significant differences in migration or cytokine secretion compared to controls.

White et al. investigated cell-extrinsic factors in systemic immune activation by using α-syn monomers and fibrils, as well as bacterial toxins, to stimulate PBMCs from PD patients and controls. They found no differences in cytokine production, nor in mRNA expression in patients vs. controls. By contrast, α-syn monomers increased production of IL-1β and IL-18 to levels significantly increased compared to those induced by low-level endotoxin.

In conclusion, this Research Topic provides a comprehensive overview of our current understanding of how adaptive and innate immune systems in the periphery are affected by infectious agents, commensal bacteria and pathogenic forms of α-syn, triggering an immune response in the central nervous system, possibly targeted at endogenous neoantigens such as α-syn itself, which eventually feeds neuroinflammation and neurodegeneration. Despite all this knowledge, however, much research is still required to establish the nature of immune dysregulation occurring in PD, how the immune system is involved in the prodromal phases of PD, and whether targeting peripheral immunity may favorably affect disease progression. We strongly hope that this collection of articles, providing a new insight of the physiopathology of PD, will encourage more laboratory and clinical research leading to the development of novel immunotherapeutics and probiotics as treatments of this disorder.

## Author Contributions

All authors listed have made a substantial, direct and intellectual contribution to the work, and approved it for publication.

### Conflict of Interest

The authors declare that the research was conducted in the absence of any commercial or financial relationships that could be construed as a potential conflict of interest.
